# Linear Polystyrene-Stabilized PdO Nanoparticle-Catalyzed Mizoroki-Heck Reactions in Water

**DOI:** 10.3390/molecules16119067

**Published:** 2011-10-27

**Authors:** Atsushi Ohtaka, Tomohiro Yamaguchi, Takuto Teratani, Osamu Shimomura, Ryôki Nomura

**Affiliations:** 1 Department of Applied Chemistry, Faculty of Engineering, Osaka Institute of Technology, 5-16-1 Ohmiya, Asahi, Osaka 535-8585, Japan; 2 Nanomaterials and Microdevices Research Center, Osaka Institute of Technology, 5-16-1 Ohmiya, Asahi, Osaka 535-8585, Japan

**Keywords:** polystyrene, PdO nanoparticles, Mizoroki-Heck reaction, water

## Abstract

Linear polystyrene-stabilized PdO nanoparticles (PS-PdONPs) were prepared by thermal decomposition of Pd(OAc)_2_ in the presence of polystyrene. X-ray diffraction (XRD) and transmission electron microscopy (TEM) indicated the production of PdO nanoparticles. The loading of palladium was determined by inductively coupled plasma-atomic emission spectroscopy (ICP-AES). PS-PdONPs exhibited high catalytic activity for Mizoroki-Heck reactions under air in water and could be recycled without loss of activity.

## 1. Introduction

The coupling reaction of vinyl or aryl halides with various alkenes in the presence of palladium catalyst is known as the Mizoroki-Heck reaction and represents a powerful tool for building up a new carbon-carbon bonds [1,2]. Recently, attention has focused on the use of palladium nanoparticles (PdNPs) as catalysts in organic synthesis [3,4,5]. PdNPs supported by a variety of substrates, including poly(*N*-vinylpyrrolidone)- or several organic moieties-grafted silica [6,7], alumina-based oxides [8], mesoporous silica or NiFe_2_O_4_ [9,10], and chitosan [11], have been shown to exhibit high catalytic activity for the Mizoroki-Heck reaction in aprotic, polar solvents such as *N*,*N*-dimethylformamide and dimethylacetamide. However, these solvents are toxic and have high boiling points, which cause difficulties in isolating the products after the reactions. This problem can be minimized by using ionic liquids [12] or performing the reaction under solvent-free conditions [13].

On the other hand, the use of water as a reaction medium for organic synthesis has recently received much attention because water is a readily available, safe, and environmentally benign solvent [14,15,16,17,18]. Several research groups have reported PdNPs-catalyzed Mizoroki-Heck reactions in water [19,20,21,22,23,24,25]. For example, Cacchi *et al.* have developed fluorous silica gel-immobilized perfluoro-tagged PdNPs that can be successfully used and recycled in the Mizoroki-Heck reaction of aryl iodides with allylic alcohols under aerobic phosphine-free conditions [26].

Recently, we found that PdO nanoparticles (PdONPs) are readily stabilized on linear polystyrene, and the resultant polystyrene-stabilized PdONPs (PS-PdONPs) have high catalytic activities for Suzuki and copper-free Sonogashira coupling reactions in water [27,28,29]. Our continuing interest in the catalytic utility of PS-PdONPs led us to examine herein the Mizoroki-Heck reaction in water.

## 2. Results and Discussion

### 2.1. Preparation and Characterization of PS-PdONPs

**Figure 1:**
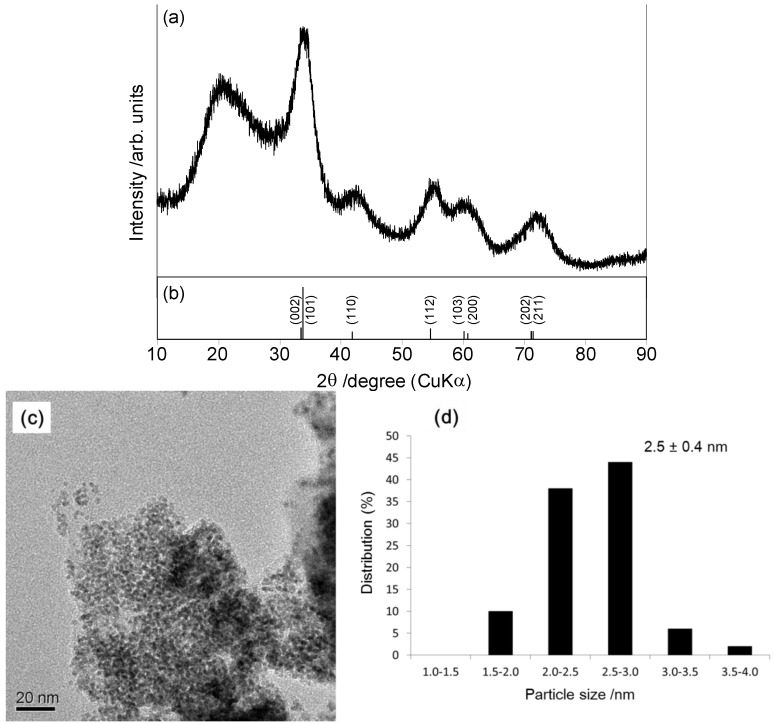
(**a**)XRD patterns of PS-PdONPs; (**b**)JCPDS data (#41-1107) for PdO; (**c**) TEM micrograph of PS-PdONPs (scale bar = 20 nm); (**d**) Size distribution of PS-PdONPs.

Linear polystyrene-stabilized PdO nanoparticles (PS-PdONPs) were prepared according to our previous paper [27]. A mixture of Pd(OAc)_2_ and linear polystyrene (*M*_n_ = 6.0 × 10^3^) was added to 1.5 mol·L^−1^ aqueous K_2_CO_3_ solution. After the mixture was stirred at 90 °C for 1 h, the color turned black. An XRD pattern of PS-PdONPs is presented in Figure 1a. In addition to the broad diffraction with 2*θ* ranging from 12° to 28° ascribed to the polystyrene, other five diffraction peaks assigned to PdO (JCPDS #41-1107) are observed clearly. Figure 1c shows a TEM image of PS-PdONPs, where a fairly uniform particle size of 2.5 ± 0.4 nm is evident. Inductively coupled plasma-atomic emission spectroscopy (ICP-AES) revealed that PS-PdONPs contained an average of 2.5 mmol·g^−1^ of Pd.

### 2.2. Coupling Conditions

**Figure 2:**
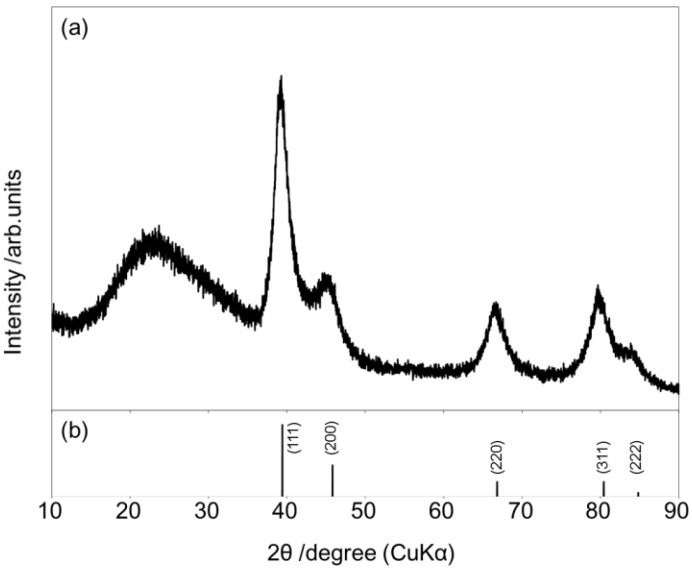
(**a**)XRD patterns of the catalyst after treatment with acrylic acid at 90 °C;(**b**)JCPDS data (#87-0641) for Pd (H-loaded).

Initially, an optimum base was investigated at 90 °C in the Mizoroki-Heck reaction of iodobenzene with acrylic acid using PS-PdONPs. As seen in Table 1, the most effective base was potassium hydroxide. Reactions with potassium carbonate, cesium carbonate, and sodium acetate gave *trans*-cinnamic acid in 27%, 19% and 20% yields, respectively, probably due to the low basicity. When 1.8-diazabicyclo[5.4.0]undec-7-ene (DBU) and NEt_3_ was used as a base, the yields of coupling product were 21% and 96%, respectively. However, the catalyst was not recovered in these cases, suggesting that the strong binding capability of nitrogen was causing palladium leaching. PS-PdNPs, reduced by NaBH_4_, exhibited slightly lower catalytic activity than PS-PdONPs although reduction of palladium on the surface of the nanoparticles was observed by XRD after treatment of PS-PdONPs with acrylic acid (Figure 2). However, the precise reason for this is as yet unclear, although it suggests that the presence of oxygen is important [27,30]. On the contrary, Pd/C exhibited low catalytic activity.

**Table 1:** Effect of base on the Mizoroki-Heck reaction of iodobenzene with acrylic acid using PS-PdONPs in water. 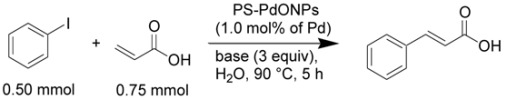

Entry	Base	Yield (%) ^a^
1	KOH	99 (32) ^b^
2 ^c^		99 (16) ^b^
3 ^d^		44
4	K_2_CO_3_	27
5	Cs_2_CO_3_	19
6	CH_3_COONa	20
7	DBU	21
8	NEt_3_	96

^a^ NMR yields; ^b^ Reaction time = 1 h; ^c^ PS-PdNPs was used as a catalyst; ^d^ Pd/C was used as a catalyst.

### 2.3. Substrate Tolerance

Employing the optimized reaction conditions, we next examined a variety of substituted aryl iodides having either an electron-releasing or an electron-withdrawing group (Table 2). The reaction of iodobenzene with acrylic acid took place smoothly in water at 90 °C for 5 h to give *trans*-cinnamic acid in 99% yield (entry 1). The Mizoroki-Heck reaction of 4-iodotoluene and 4-iodoanisole, bearing electron donating groups at the *para*-position, gave the corresponding cinnamic acids in 99% and 96% yields, respectively (entries 2 and 3). Substrates with electron-deficient aromatic rings, *i.e.*, 4-iodoacetophenone and 4-iodobenzotrifluoride, also underwent the Mizoroki-Heck reaction with acrylic acid under similar conditions to afford 4-acetylcinnamic acid and 4-trifluoromethylcinnnamic acid, respectively, both in near quantitative yield (entries 4 and 5). Sterically hindered substrates were also examined. The reaction of 2-iodotoluene and 2-iodophenol with acrylic acid gave the corresponding cinnamic acids in 99% and 99% yields, respectively (entries 6 and 7). 1-Iodonaphthalene was also reactive, with the desired product being obtained in 98% yield (entry 8). It is noteworthy that the formation of 2,6-dimethylcinnamic acid was achieved by the reaction of 2-iodo-*m*-xylene with acrylic acid in 59% yield (entry 9). The reaction proceeded well with 4-bromoacetophenone, although a longer reaction time was needed (entry 4). However, reactions with styrene and bromobenzene gave low yields (entries 1 and 10).

**Table 2:** PS-PdONPs-catalyzed Mizoroki-Heck reaction in water. 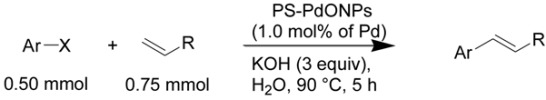

Entry	Aryl iodides	Alkenes	Yield (%) ^a^
1	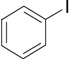	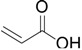	99 (13) ^b,c^
2	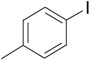	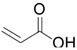	99
3	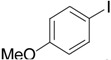	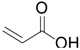	96
4	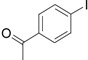	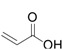	99 (99) ^b,c^
5	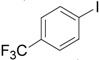	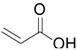	92
6	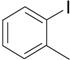	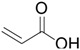	99
7	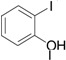	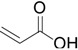	99
8	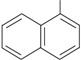	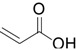	98
9	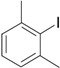	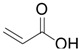	59 ^c^
10	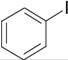	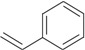	14 ^c^

^a^ NMR yields; ^b^ Aryl bromide was used as a substrate; ^c^ Reaction time = 20 h.

### 2.4. Recycling Experiments

**Scheme 1:**
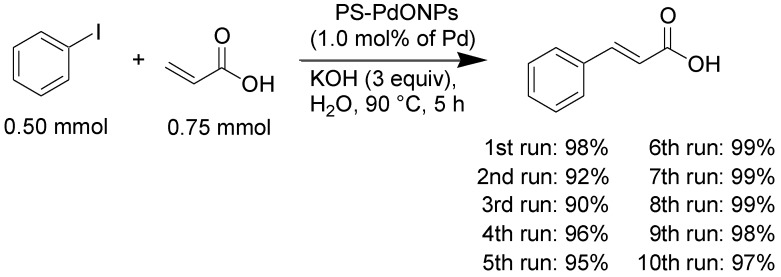
Recycling experiments.

**Figure 3:**
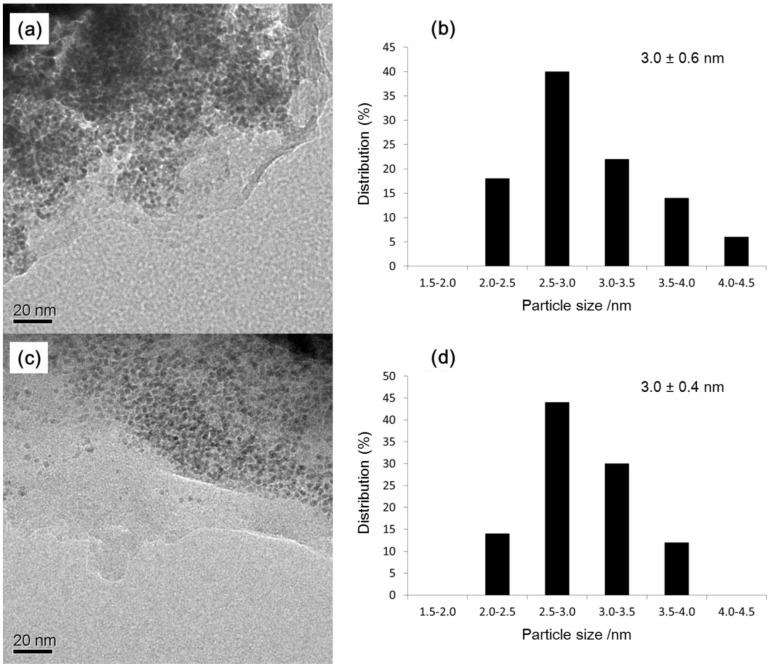
(**a**) TEM image of the recovered catalyst after the fifth run; (**b**)Size distribution of the recovered catalyst after the fifth run; (**c**) TEM image of the recovered catalyst after the tenth run; (**d**)Size distribution of the recovered catalyst after the tenth run.

Recycling studies were then performed. After the first reaction, which gave a nearly quantitative yield of the product (Table 2, entry 1), the catalyst was recovered and successively subjected to nine more runs of the reaction under the same conditions. As shown in Scheme 1, the yields remained essentially constant for the ten successive runs. After every run, the reaction solutions were analyzed by inductively coupled plasma-atomic emission spectroscopy (ICP-AES) to determine the amount of palladium leached during the reaction. The amount of palladium leaching after every run was <1.4%. Similar sizes of palladium nanoparticles were observed by TEM after the recycling experiments (after the fifth run, 3.0 ± 0.6 nm; after the tenth run, 3.0 ± 0.4 nm, Figure 3). When the reaction was interrupted at 16% conversion and continued after removal of the catalyst (hot filtration test), the residual activity of the reaction mixture was significant (47% after 20 h). This suggests that leached palladium species are, obviously, participating in the catalytic process. However, the data in Table 1 (entries 1 and 2) and the hot filtration test indicate that the soluble forms of palladium are not the only catalytically active species.

## 3. Experimental

### 3.1. General

^1^H-NMR spectra in CDCl_3_ were recorded with a 300 MHz NMR spectrometer (UNITY 300, Varian, Palo Alto, CA, USA) using tetramethylsilane (δ = 0) as an internal standard. Inductively coupled plasma-atomic emission spectroscopy (ICP-AES) was performed using ICPS-8100 (Shimadzu Co., Kyoto, Japan). Pd nanoparticles were investigated by transmission electron microscopy (TEM) on a JEM 2100F transmission electron microscope (JEOL Ltd., Tokyo, Japan). The samples were prepared by placing a drop of the solution on carbon-coated copper grids and allowed to dry in air. Polystyrene of narrow molecular weight distribution standards was purchased from Tosoh Co., Ltd. (Tokyo, Japan). Pd(OAc)_2_ was obtained from Sigma-Aldrich Co. (St Louis, MI, USA).

### 3.2. Preparation of PS-PdONPs

To a screw-capped vial with a stirring bar was added polystyrene (9.0 mg, 85 μmol of styrene units), Pd(OAc)_2_ (5.5 mg, 25 μmol), and 1.5 M aqueous K_2_CO_3_ solution (3 mL). After stirring at 90 °C for 1 h, the reaction mixture was filtered with hot water. Subsequently, the polystyrene stabilized Pd nanoparticles were washed with hot water (5 × 1.0 mL) and acetone (5 × 1.0 mL).

### 3.3. Determination of the Amount of Palladium

PS-PdONPs (2.9 mg) was placed in a screw-capped vial and then 13 M nitric acid (5 mL) was added. The mixture was heated at 80 °C to dissolve completely. After cooling to room temperature, the solution was adjusted to 50 g by water and then the amount of Pd metal was measured by ICP-AES analysis (15.3 ppm). After the catalytic reaction, the aqueous phase was adjusted to 10 g by nitric acid and then the amount of Pd metal was measured by ICP-AES analysis.

### 3.4. Typical Procedures for Mizoroki-Heck Reaction

To a screw-capped vial with a stirring bar were added iodobenzene (0.25 mmol), acrylic acid (0.25 mmol), PS-PdONPs (1.0 mol% of Pd), 1.5 M aqueous KOH solution (1 mL). After stirring at 90 °C for 5 h, the reaction mixture was cooled to room temperature by immediately immersing the vial in water (~20 °C). Subsequently, the aqueous phases were removed, and recovered catalyst was washed with water (5 × 1.5 mL) and diethyl ether (5 × 1.5 mL), which were then added to the aqueous phase. After 6.0 mol·L^−1^ HCl aqueous solution (0.22 mL) was added to the aqueous phase, the aqueous phase was extracted five times with diethyl ether. The combined organic extracts were dried over MgSO_4_, concentrated under reduced pressure. The product was analyzed by ^1^H-NMR. The recovered catalyst was dried *in vacuo* and successfully reused. Furthermore, the amount of Pd metal in the aqueous phase determined by ICP-AES analysis was 0.1 ppm.

## 4. Conclusions

PS-PdONPs was prepared with a simple procedure and demonstrated to be an efficient and reusable catalyst for the Mizoroki-Heck reaction in water. ICP-AES analysis confirmed that palladium leached into the aqueous solution during the reaction. Hot filtration tests indicated the leached palladium species are participating in the catalytic process. In addition, no obvious change in particle size was observed by TEM. Currently, further efforts to extend the application of polystyrene-stabilized metal nanoparticles to other organic reaction in water are under way in our laboratory.
